# Dr. Marshall Hall

**Published:** 1853-06

**Authors:** 


### DR. MARSHALL HALL.

This distinguished physician visited our city a month since and gratified a number of our professional brethren by performing in their presence a series of experiments to illustrate the functions of the spinal cord, of which our readers have been informed by our correspondent, Dr. Wible. We were absent from the city at the time and consequently had not the pleasure of witnessing these experiments. Dr. Hall has been pressed by his friends to return to Louisville in the autumn or winter, to deliver a course ot lectures on the physiology and pathology of the Spinal System, and, we trust, will gratify their wishes.

